# Human Respiratory Syncytial Virus NS2 Protein Induces Autophagy by Modulating Beclin1 Protein Stabilization and ISGylation

**DOI:** 10.1128/mbio.03528-21

**Published:** 2022-01-18

**Authors:** Kim Chiok, Swechha M. Pokharel, Indira Mohanty, Lindsay Grace Miller, Shou-Jiang Gao, Arthur L. Haas, Kim C. Tran, Michael N. Teng, Santanu Bose

**Affiliations:** a Department of Veterinary Microbiology and Pathology, College of Veterinary Medicine, Washington State University, Pullman, Washington, USA; b Cancer Virology Program, UPMC Hillman Cancer Center, Department of Microbiology and Molecular Genetics, University of Pittsburgh, Pittsburgh, Pennsylvania, USA; c Department of Biochemistry and Molecular Biology, Louisiana State University School of Medicine, New Orleans, Louisiana, USA; d Division of Allergy and Immunology, Department of Internal Medicine, University of South Florida Morsani College of Medicine, Tampa, Florida, USA; Princeton University

**Keywords:** autophagy, paramyxovirus, respiratory syncytial virus

## Abstract

Paramyxoviruses such as respiratory syncytial virus (RSV) are the leading cause of pneumonia in infants, the elderly, and immunocompromised individuals. Understanding host-virus interactions is essential for the development of effective interventions. RSV induces autophagy to modulate the immune response. The viral factors and mechanisms underlying RSV-induced autophagy are unknown. Here, we identify the RSV nonstructural protein NS2 as the virus component mediating RSV-induced autophagy. We show that NS2 interacts and stabilizes the proautophagy mediator Beclin1 by preventing its degradation by the proteasome. NS2 further impairs interferon-stimulated gene 15 (ISG15)-mediated Beclin1 ISGylation and generates a pool of “hypo-ISGylated” active Beclin1 to engage in functional autophagy. Studies with NS2-deficient RSV revealed that NS2 contributes to RSV-mediated autophagy during infection. The present study is the first report to show direct activation of autophagy by a paramyxovirus nonstructural protein. We also report a new viral mechanism for autophagy induction wherein the viral protein NS2 promotes hypo-ISGylation of Beclin1 to ensure availability of active Beclin1 to engage in the autophagy process.

## INTRODUCTION

Respiratory syncytial virus (RSV) is a leading cause of viral pneumonia in infants, the elderly, and immunocompromised patients. For the year 2015, approximately 3.2 million hospital admissions due to acute low respiratory tract infection in children younger than 5 years were attributed to RSV worldwide (1). Clinically approved and effective therapeutic options are scarce, with prophylactic monoclonal antibodies (i.e., palivizumab) (2) and antiviral compounds such as ribavirin showing variable clinical outcomes (3). No licensed vaccine is currently available. Therefore, understanding host-virus interactions and mechanisms governing the ability of RSV to alter the immune response is essential for the development of effective therapeutic and prophylactic interventions.

An emerging branch of evidence shows that innate immune responses are regulated by a process known as autophagy. Autophagy is at the center of cellular homeostasis, as this process degrades damaged proteins and organelles in specialized enclosed compartments. The by-products of such degradation are then recycled by the cell, a feature useful during stress and nutrient starvation under which autophagy preserves cell viability (reviewed in reference 4). The autophagy pathway comprises a network of interacting protein complexes that drive the formation of membranous compartments known as autophagosomes (5). The autophagy core complex consists of VPS15-VPS34-ATG14-Beclin1, which reorganizes membranes derived from cytoplasmic organelles, converting them into the phagophore and autophagosome that ultimately drive autophagy. Autophagy can be regulated by posttranslational modifications in its core complex proteins. For instance, recent work indicates that the proautophagy mediator Beclin1 is subject to ISGylation, resulting in inhibition of autophagy (6). ISGylation entails the covalent conjugation of interferon-stimulated gene 15 (ISG15) to target proteins (7, 8) as a means to regulate a wide spectrum of cellular activities, including autophagy. To date, there are no reports of autophagy regulation by viral proteins through modulation of ISGylation. In the current study, we show that RSV NS2 protein deregulates ISGylation of Beclin1 to promote autophagy during infection.

Autophagy participates in immune responses against virus infections by degrading viral proteins and blocking virus spread (9), activating and signaling Toll-like receptors (TLRs) (10), and mediating secretion of immune modulators (11) and cytokines (10), among numerous others. RSV infection induces autophagy in cultured lung epithelial cells, mouse airway cells, dendritic cells, and macrophages (10, 12–14). RSV-induced autophagy contributes to maturation and cytokine expression in dendritic cells (12) and activation of the TGF-β/SMAD signaling pathway in macrophages (14). Despite the various roles of autophagy during RSV infection, the mechanisms responsible for triggering RSV-induced autophagy remain unknown. Specifically, the role of any viral component in inducing autophagy during RSV infection has not yet been identified. Here, we identify RSV nonstructural protein 2 (NS2) as a key regulator of autophagy during infection.

Single-stranded negative-sense RNA viruses encode several nonstructural proteins. Orthomyxoviruses (e.g., influenza viruses) encode NS1, while paramyxoviruses encode nonstructural proteins such as NS1 and NS2 (RSV), C protein (human parainfluenza 3, measles, Nipah, and Hendra viruses), V protein (human parainfluenza 3, mumps, measles, Nipah, and Hendra viruses), and W protein (measles, Nipah, and Hendra viruses). These proteins are not packaged in the virus particle and are expressed only in infected cells. Nonstructural proteins of single-stranded negative-sense RNA viruses antagonize the innate immune response by interfering with type I interferon signaling and inflammasome-mediated interleukin-1beta (IL-1β) production. To date, the role of RSV nonstructural proteins in autophagy remains unexplored.

Our current study highlights a new role of nonstructural proteins by showing that RSV NS2 mediates RSV-induced autophagy. Our data indicate that NS2 induces autophagy by interacting with the proautophagy mediator Beclin1, preventing its degradation and leading to enhanced Beclin1 protein levels and functional autophagy. Surprisingly, NS2 also hinders ISGylation of Beclin1, partially relieving the antiautophagy effect of ISGylated Beclin1 and enabling Beclin1 to proceed with autophagy. Studies with NS2-deficient RSV have revealed that NS2 contributes to RSV-mediated autophagy during infection. Thus, our current study identifies RSV NS2 as a proautophagic viral factor and provides a mechanistic model for RSV-induced autophagy in which NS2 employs a dual approach: increase Beclin1 protein levels by preventing its degradation and relieve it from ISGylation to trigger autophagy.

## RESULTS

### RSV NS2 induces autophagy.

The paramyxovirus respiratory syncytial virus (RSV) induces autophagy in mouse lung tissue, lung epithelial cells, macrophages (14), and dendritic cells (10, 15). However, the viral components and molecular mechanisms underlying RSV-induced autophagy remain undefined. We employed the widely used RSV HEp-2 and A549 cell models (16–18) to identify the viral components that mediate RSV-induced autophagy. We investigated potential roles for RSV’s nonstructural (NS) proteins NS1 and NS2 in autophagy induction because of their extensive ability to manipulate the immune response (reviewed in reference 19).

**FIG 1:**
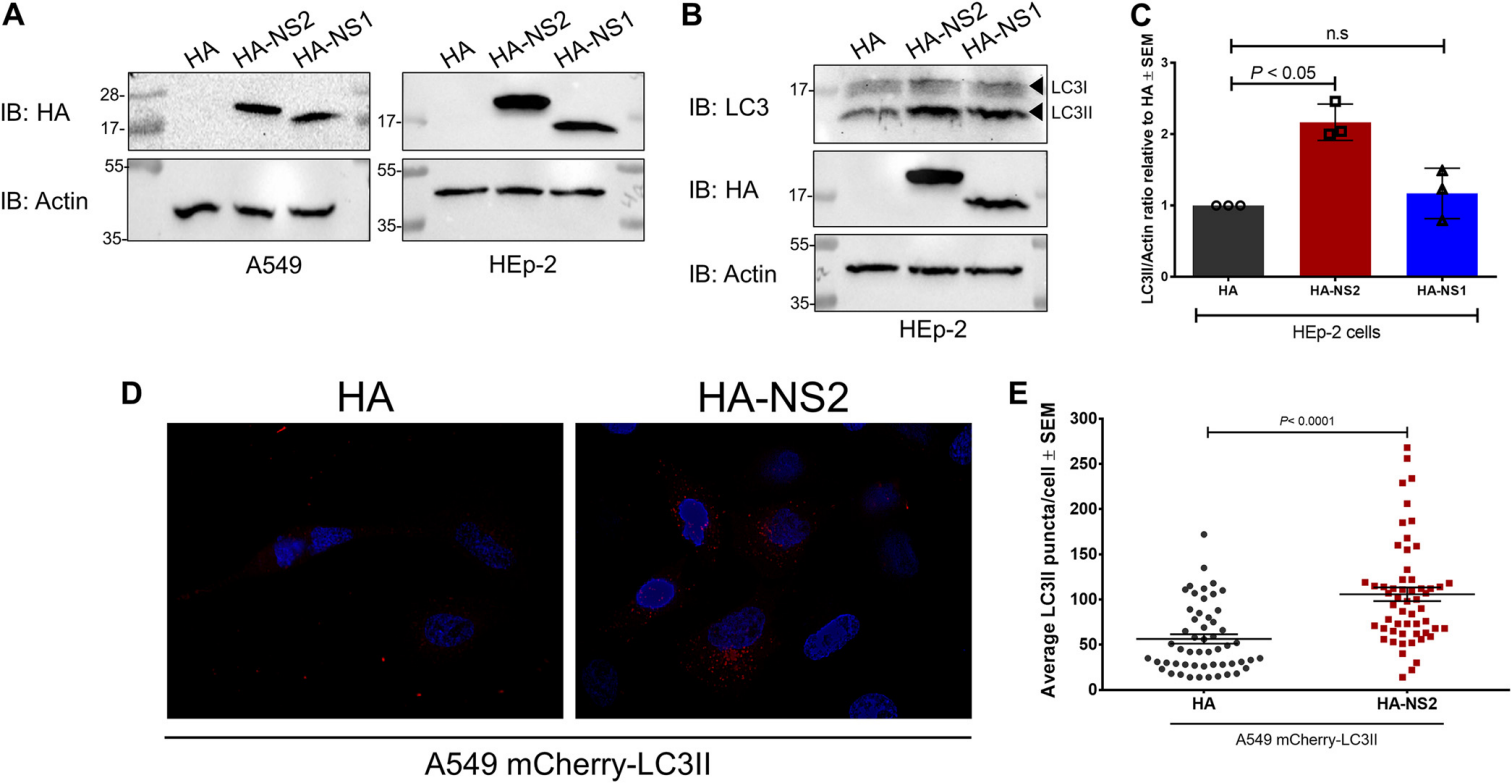
RSV NS2 protein induces autophagy. (A) A549 and HEp-2 cells were transfected with empty HA vector control (HA), HA-tagged NS2 (HA-NS2), or HA-tagged NS1 (HA-NS1) for 12 h. Expression was evaluated by Western blot analysis using anti-HA antibody. (B and C) Western blotting (B) and densitometry analysis (C) of LC3II and LC3I protein levels in HEp-2 cells expressing vector control (HA), HA-NS2, or HA-NS1. (D) A549 cells stably expressing mCherry-LC3II were transfected with vector control (HA) or HA-NS2 for 16 h. LC3II puncta formation was assessed by fluorescence microscopy. (E) Images captured from panel D were used to quantify the number of LC3II puncta per cell (*n* = 25 cells per experiment, 2 independent experiments) using ImageJ software. Immunoblot images are representative of three independent experiments. One-way ANOVA followed by Dunnett’s test and Student’s *t* test were performed to evaluate results derived from Western blot densitometry analysis and fluorescence microscopy analysis, respectively. n.s, not significant.

We first transfected plasmids encoding hemagglutinin (HA)-tagged RSV NS1 and NS2 proteins into A549 and HEp-2 cells and confirmed the expression of NS1 and NS2 in these cells (Fig. 1A). To examine the roles of nonstructural proteins in RSV-mediated autophagy, we monitored the levels of the autophagy marker LC3II (20, 21) after expression of NS1 and NS2 in HEp-2 cells by immunoblotting (IB) (Fig. 1B). Densitometry analysis of the LC3II/actin ratio from immunoblots revealed that expression of NS2 resulted in significantly increased levels of LC3II, reaching up to twice the levels observed in cells expressing vector alone (*P < *0.05) (Fig. 1C). In contrast, NS1 failed to significantly enhance LC3II levels in HEp-2 cells (Fig. 1C). Similar upregulation of autophagy by NS2 was observed in A549 cells (see Fig. S1 in the supplemental material).

10.1128/mbio.03528-21.1 FIG S1 RSV NS2 protein induces autophagy in A549 cells. Western blot analysis of LC3II and LC3I protein levels in A549 cells expressing vector control (HA), HA-NS2, or HA-NS1. Download FIG S1, PDF file, 0.1 MB. Copyright © 2022 Chiok et al. 2022 Chiok et al. https://creativecommons.org/licenses/by/4.0/ This content is distributed under the terms of the Creative Commons Attribution 4.0 International license.

In addition to enhanced LC3II levels, an increase in numbers of characteristic LC3II puncta is an indicator of enhanced autophagy, as LC3II is incorporated into autophagosomes, which can be examined by microscopy in cells expressing fluorescent LC3II (20, 21). Therefore, we examined the appearance and number of LC3II puncta in A549 cells expressing NS2 and mCherry-LC3II to ascertain autophagy status by using fluorescence microscopy (22). Expression of NS2 led to the formation of typical LC3II puncta in the cytoplasm of A549 cells (Fig. 1D). Quantification of LC3II puncta revealed significantly (*P < *0.0001) increased LC3II puncta per cell for those expressing NS2 compared to the control (Fig. 1E). Moreover, inhibition of autophagic flux by bafilomycin A resulted in further accumulation of LC3II in cells expressing NS2 compared to untreated cells (Fig. S2). These data indicate that NS2 does not block autophagy flux, and thus, enhanced LC3II levels in the presence of NS2 are not due to functional defects in autophagy. Therefore, expression of NS2 led to increased abundance of the autophagy marker LC3II (Fig. 1B and C; Fig. S1) and its subsequent incorporation into autophagosomes (Fig. 1D and E). These results indicate that RSV NS2 can directly induce autophagy.

10.1128/mbio.03528-21.2 FIG S2 NS2 protein induces autophagy. (A) HEp-2 cells were transfected with empty vector control (HA) or HA-tagged NS2 (HA-NS2) and treated with methanol (vehicle control) or bafilomycin A1 (BafA1; 30 nM) for 24 h. Autophagy status was determined by Western blot analysis of LC3II and LC3I protein levels. (B) Densitometry analysis of LC3II levels relative to actin (LC3II/actin) in HEp-2 cells expressing vector control (HA) or HA-NS2 and treated with either BafA1 or vehicle control. Error bars represent the standard error of the mean (SEM) of results from 3 biologically independent experiments. *, *P < *0.05 (determined by one-way ANOVA adjusted for multiple comparisons by Dunnett’s *post hoc* test). IB, immunoblotted. Download FIG S2, PDF file, 0.1 MB. Copyright © 2022 Chiok et al. 2022 Chiok et al. https://creativecommons.org/licenses/by/4.0/ This content is distributed under the terms of the Creative Commons Attribution 4.0 International license.

### NS2 interacts with Beclin1.

To dissect the mechanism underlying NS2-mediated autophagy, we examined potential interactions between NS2 and Beclin1, given the proautophagy role of Beclin1. Beclin1 is a fundamental positive regulator of autophagy via its interaction with other core proteins to mediate assembly of the VPS34-VPS15-Beclin1-ATG14 complex during autophagy initiation (reviewed in reference 23). Expression of NS2 enhanced the levels of autophagy marker LC3II and LC3II incorporation into autophagosomes, indicating that NS2 can induce autophagy (Fig. 1B to E; Fig. S1 and S2).

**FIG 2:**
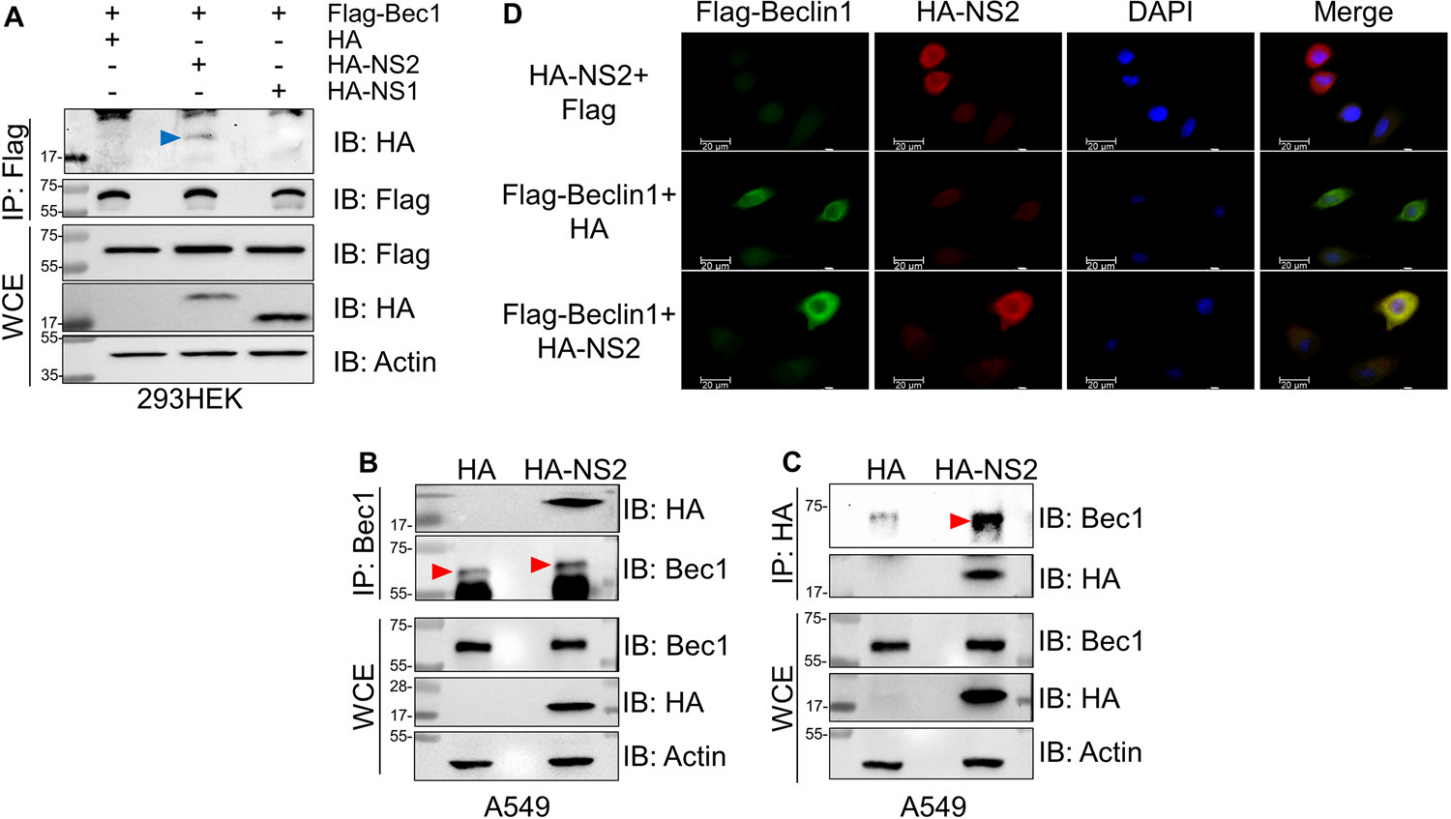
RSV NS2 protein interacts with Beclin1 protein. (A) Coimmunoprecipitation analysis of 293HEK cells coexpressing Flag-tagged Beclin1 (Flag-Bec1) and either HA-tagged NS2 (HA-NS2), HA-tagged NS1 (HA-NS1), or empty HA vector control (HA). Beclin1 was immunoprecipitated (IP) with anti-Flag antibody, followed by immunoblotting (IB) with either anti-HA or anti-Flag antibody. Whole-cell extracts (WCE) were immunoblotted with anti-Flag, anti-HA, and anti-actin antibodies. A blue arrowhead indicates NS2 protein. (B) Coimmunoprecipitation analysis of A549 cells expressing HA-NS2. Endogenous Beclin1 was IP with anti-Beclin1 antibody, followed by IB with either anti-HA or anti-Beclin1 antibody. WCE were immunoblotted with anti-HA and anti-actin antibodies. Red arrowheads indicate Beclin1 protein. (C) Coimmunoprecipitation analysis of A549 cells expressing HA-NS2. HA-NS2 was IP with anti-HA antibody, followed by IB with either anti-HA or anti-Beclin1 antibody. WCE were immunoblotted with anti-HA and anti-actin antibodies. A red arrowhead indicates Beclin1 protein. (D) Coimmunofluorescence analysis of Beclin1 and NS2 interaction in A549 cells coexpressing Flag-Beclin1 and HA-NS2. Fixed cells were probed with either anti-Flag or anti-HA primary antibodies and subsequently labeled with fluorescent secondary antibodies: FITC (green) for Flag-Beclin1 and Alexa Fluor 594 (red) for HA-NS2. The yellow color in the merge panels indicates colocalization of NS2 with Beclin1. Immunoblot images are representative of three independent experiments. Coimmunofluorescence images are representative of 10 different fields.

First, we determined whether NS2 interacts with Beclin1 in transfected HEK293 cells expressing Flag-tagged Beclin1 and HA-tagged NS2, NS1, or vector control (HA-tagged empty vector) by coimmunoprecipitation. NS2 coprecipitated with Beclin1 (Fig. 2A), indicating that NS2 interacts with this proautophagy regulator. In accordance with the inability of NS1 to induce autophagy (Fig. 1C), we failed to detect an interaction of Beclin1 with NS1 protein (Fig. 2A). We further validated this NS2-Beclin1 interaction by reciprocal coimmunoprecipitation of NS2 and endogenous Beclin1 from A549 cells expressing HA-tagged NS2. Immunoprecipitation (IP) with anti-Beclin1 antibody followed by immunoblotting with anti-HA antibody revealed an interaction of NS2 with endogenous Beclin1 (Fig. 2B). Conversely, immunoprecipitation with anti-HA antibody followed by immunoblotting with Beclin1 antibody further confirmed the NS2-Beclin1 interaction (Fig. 2C). Similar results were obtained with HEp-2, wherein HA-NS2 also interacted with endogenous Beclin1 (Fig. S3). These results show that NS2 interacts with the autophagy mediator Beclin1.

10.1128/mbio.03528-21.3 FIG S3 RSV NS2 protein interacts with Beclin1 protein in HEp-2 cells. (A) Coimmunoprecipitation analysis of HEp-2 cells expressing either HA-tagged NS2 (HA-NS2) or empty HA vector control (HA). Endogenous Beclin1 (Bec1) was immunoprecipitated (IP) with anti-Beclin1 antibody, followed by immunoblotting (IB) with either anti-HA or anti-Beclin1 antibody. WCE were IB with anti-HA, anti-Beclin1, and anti-actin antibodies. A red arrowhead indicates the RSV NS2 protein coimmunoprecipitating with Beclin1 protein. (B) Coimmunoprecipitation analysis of HEp-2 cells expressing either HA-NS2 or empty HA vector control (HA). HA-NS2 was IP with anti-HA antibody, followed by IB with either anti-HA or anti-Beclin1 antibody. WCE were immunoblotted with anti-HA, anti-Beclin1, and anti-actin antibodies. A red arrowhead indicates Beclin1 protein coimmunoprecipitating with RSV NS2 protein. Download FIG S3, PDF file, 0.2 MB. Copyright © 2022 Chiok et al. 2022 Chiok et al. https://creativecommons.org/licenses/by/4.0/ This content is distributed under the terms of the Creative Commons Attribution 4.0 International license.

To confirm that the NS2-Beclin1 interaction occurs in intact cells, we next investigated whether NS2 colocalized with Beclin1 in lung epithelial cells. A549 cells coexpressing HA-tagged NS2 and Flag-tagged Beclin1 were subjected to immunofluorescence microscopy to determine the intracellular localization of these proteins. Both Beclin1 (green) and NS2 (red) were localized in the cytoplasm when expressed individually (Fig. 2D). Coexpression of NS2 and Beclin1 resulted in their colocalization in the cytoplasm, as evidenced by the yellow color in merged images in Fig. 2D. These results demonstrate that RSV NS2 protein interacts and colocalizes with the autophagy-inducing Beclin1 protein.

### NS2 interacts with the BH3, CCD, and ECD domains of Beclin1.

**FIG 3:**
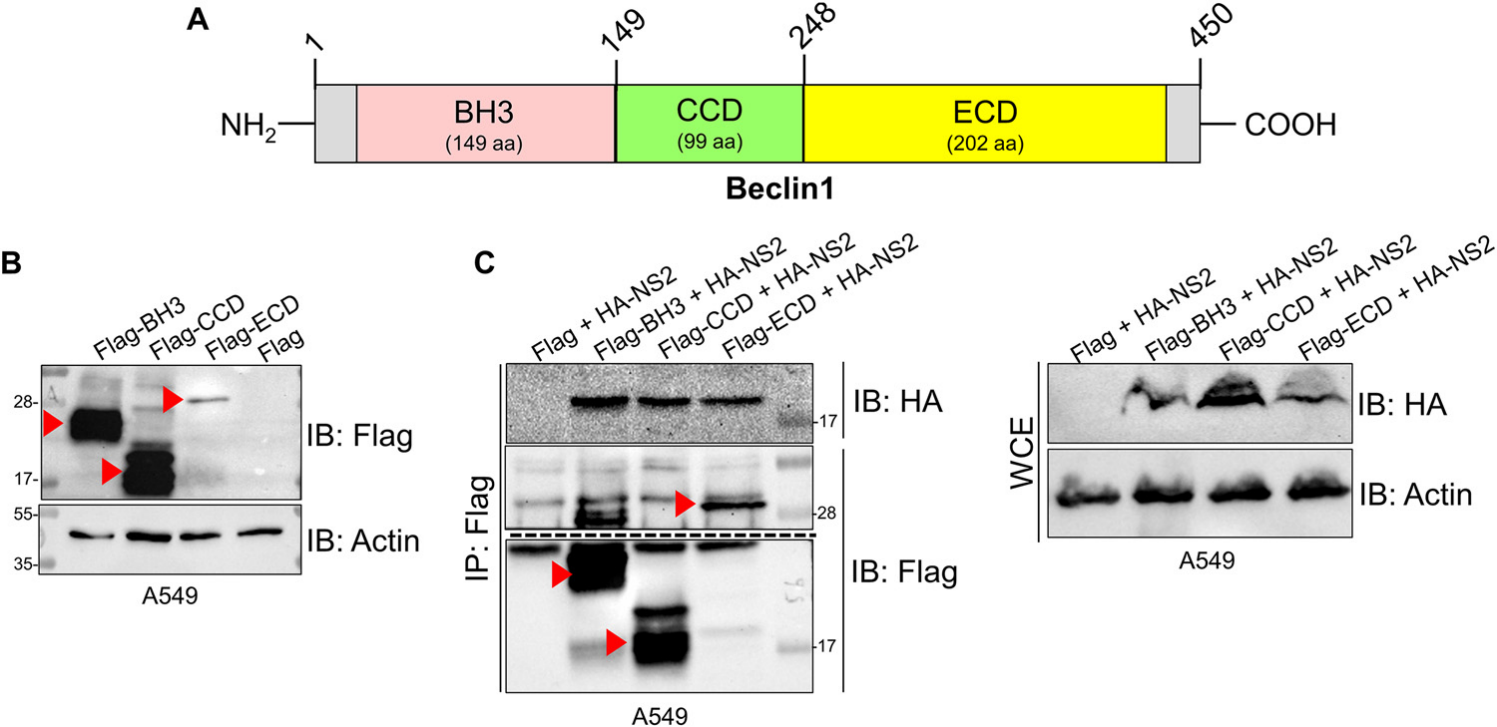
RSV NS2 protein interacts with the domains BH3, CCD, and ECD of Beclin1 protein. (A) Schematic diagram of functional domains BH3, CCD, and ECD of human Beclin1. aa, amino acids. (B) A549 cells were transfected with either Flag-tagged BH3 (Flag-BH3), Flag-tagged CCD (Flag-CCD), Flag-tagged ECD (Flag-ECD), or empty Flag vector control (Flag). Expression of transfected proteins was assessed in whole-cell extracts (WCE) by immunoblotting (IB) with anti-Flag antibody. Red arrowheads indicate BH3, CCD, and ECD domains of Beclin1 protein. (C) Coimmunoprecipitation analysis of A549 cells coexpressing HA-tagged NS2 (HA-NS2) and Flag-BH3, Flag-CCD, Flag-ECD, or control vector (Flag). Beclin1 domains were immunoprecipitated (IP) with anti-Flag antibody, followed by IB with either anti-HA or anti-Flag antibody. WCE were immunoblotted with anti-HA and anti-actin antibodies. Red arrowheads indicate BH3, CCD, and ECD domains of Beclin1 protein. Immunoblot images are representative of three independent experiments.

Since NS2 interacted and colocalized with Beclin1, we aimed to determine which Beclin1 domains are involved in this interaction. Beclin1 consists of three functionally distinct domains: BH3, the coiled-coil domain (CCD), and the evolutionary conserved domain (ECD) (Fig. 3A). The BH3 domain determines the activation status of Beclin1 via interaction with autophagy inhibitory proteins such as BclII (24) and Bcl-XL (25, 26). The CCD is involved in the interaction of Beclin1 with UVRAG and ATG14 (27) proteins to form the autophagy core complex. The ECD mediates the association of Beclin1 with the autophagy core protein Vps34 (28). The ECD is also necessary for autophagic vesicle formation (29).

We first confirmed the expression of each of the Beclin1 domains by transfecting A549 cells with Flag-tagged BH3, CCD, and ECD, followed by immunoblotting with an anti-Flag antibody (Fig. 3B). Although we observed high expression of BH3 and CCD, expression of ECD was comparatively lower, in accordance with a previous report (30). Next, A549 cells coexpressing NS2 and each of the individual Beclin1 domains (BH3, CCD, or ECD) were subjected to coimmunoprecipitation assays to identify the Beclin1 domain(s) interacting with NS2. Interestingly, NS2 coprecipitated with each of the three domains (Fig. 3C), indicating that NS2 interacts with multiple sites on Beclin1. To determine whether the interaction of NS2 with Beclin1 domains is specific, we performed coimmunoprecipitation experiments with cells expressing HA-NS2 and Flag-tagged C-terminal domain of RIG-I protein (RIG-I-C). As expected, HA-NS2 interacted with Flag-tagged Beclin1 BH3 domain (Fig. S4). In contrast, HA-NS2 failed to interact with Flag-tagged RIG-I-C (Fig. S4). Therefore, the interaction of FLAG-tagged Beclin1 domains with HA-NS2 is specific, since HA-NS2 failed to interact with a nonspecific FLAG-tagged protein (i.e., FLAG-tagged RIG-I-C) (Fig. S4).

10.1128/mbio.03528-21.4 FIG S4 Specific interaction of RSV NS2 protein with the BH3 domain of Beclin1 protein. A549 cells were transfected with either Flag-tagged-BH3 (Flag-BH3), Flag-tagged RIG-I-C (Flag-tagged C-terminal domain of RIG-I protein), or empty Flag vector control (Flag). Flag-tagged proteins were immunoprecipitated (IP) with anti-Flag antibody, followed by immunoblotting (IB) with either anti-HA or anti-Flag antibody. Whole-cell extracts (WCE) were immunoblotted with anti-Flag, anti-HA, and anti-actin antibodies. A red arrowhead indicates the RSV NS2 protein coimmunoprecipitating with Flag-BH3. Download FIG S4, PDF file, 0.2 MB. Copyright © 2022 Chiok et al. 2022 Chiok et al. https://creativecommons.org/licenses/by/4.0/ This content is distributed under the terms of the Creative Commons Attribution 4.0 International license.

### RSV enhances Beclin1 protein levels during infection.

**FIG 4:**
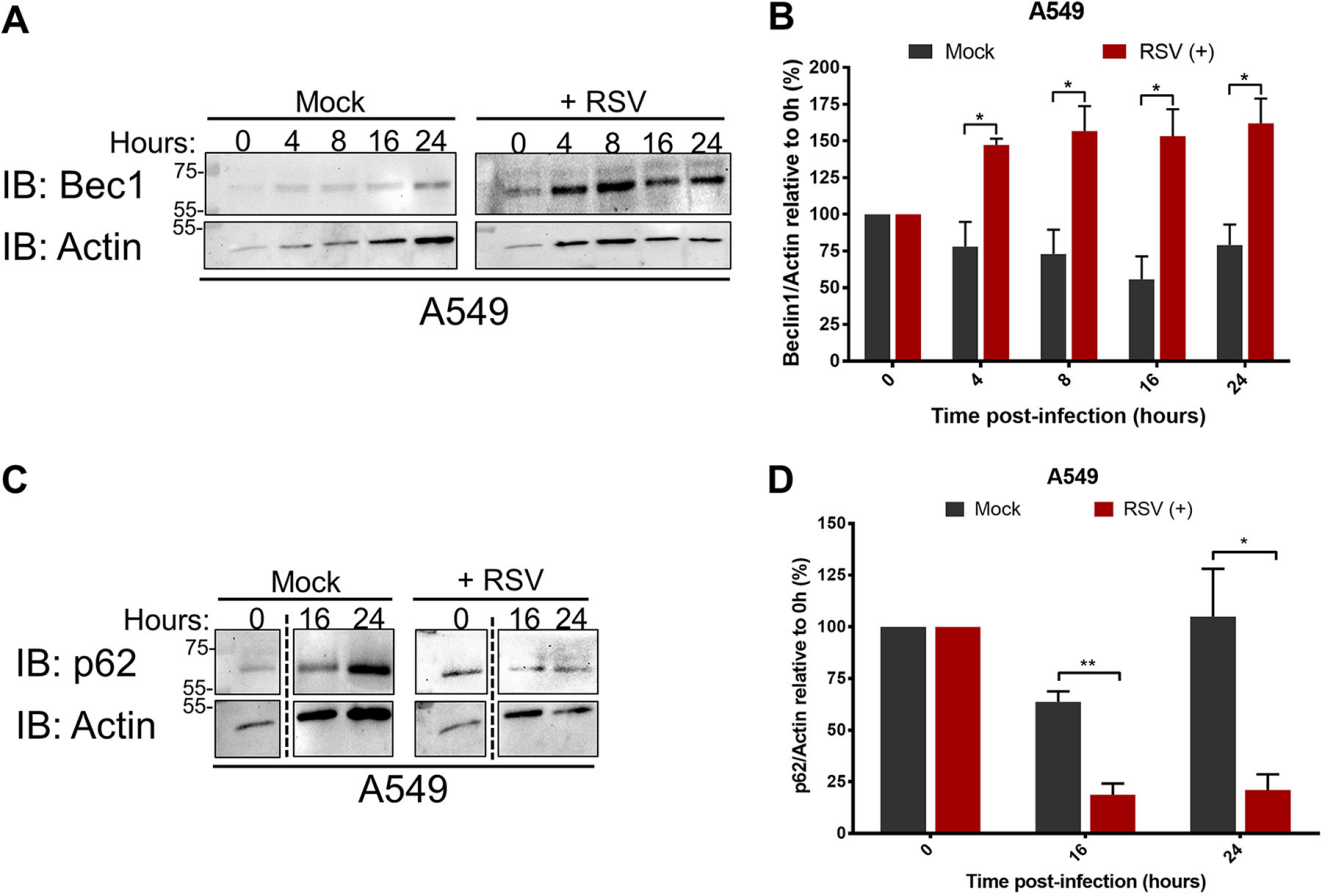
RSV enhances Beclin1 protein levels during infection. (A) A549 cells were mock infected or RSV infected (MOI = 1) for 24 h. Beclin1 (Bec1) and actin levels were determined in cell lysates by immunoblotting (IB) using corresponding antibodies. (B) Densitometry analysis of Beclin1 levels relative to actin (Beclin1/actin) in mock-infected or RSV-infected (MOI = 1) A549 cells. Beclin1/actin ratios were normalized to the ratio at 0 h of infection. (C) A549 cells were mock infected or RSV infected (MOI = 1) for 24 h. p62 and actin levels were determined in cell lysates by immunoblotting using corresponding antibodies. (D) Densitometry analysis of p62 levels relative to actin (p62/actin) in mock-infected or RSV-infected (MOI = 1) A549 cells. p62/actin ratios were normalized to the ratio at 0 h of infection. Immunoblot images are representative of three independent experiments. Error bars represent the standard error of the mean (SEM) of results from 3 biologically independent experiments. *, *P < *0.05; **, P < 0.01 (determined by Student’s *t* test adjusted for multiple comparisons by the Sidak-Bonferroni method).

We next determined the status of Beclin1 protein during RSV infection to elucidate the role of NS2 and its interaction with Beclin1 in autophagy induction. NS2 is one of the earliest and most abundant viral proteins produced during RSV infection due to its proximity to the 3′-end promoter in the RSV genome (31, 32). A549 cells were either mock infected or infected with RSV for up to 24 h to monitor Beclin1 protein levels by immunoblotting with an anti-Beclin1 antibody. RSV infection resulted in increased Beclin1 protein levels as early as 4 h postinfection compared to mock-infected cells, consistent with early NS2 production (Fig. 4A). Densitometry analysis revealed significantly enhanced (by more than 50%) Beclin1 protein levels in RSV-infected cells than in mock controls (*P < *0.05) (Fig. 4B). In contrast to Beclin1 protein, RSV infection did not upregulate *beclin1* mRNA (Fig. S5), indicating that changes in Beclin1 protein levels are not due to transcriptional upregulation. It is noteworthy that Beclin1 protein levels were higher in RSV-infected cells than in mock-infected cells at 0 h, a time point that represents a postadsorption stage following infection with RSV for 1.5 h, again consistent with early production of NS2 protein in infected cells due to the proximity of the NS2 gene near the RSV genome transcription start site (i.e., 3′ end of the RSV genome). Increased Beclin1 protein levels have been associated with induction of autophagy (33, 34). Our results suggest that NS2 protein expressed during RSV infection enhances Beclin1 protein levels to induce autophagy.

10.1128/mbio.03528-21.5 FIG S5 The transcript level of *beclin1* is not upregulated during RSV infection. RNA from A549 cells mock infected (−RSV) or infected with RSV at an MOI of 1 (A) or 3 (B) was subjected to RT-qPCR to determine *beclin1* transcript levels. The average expression relative to that of mock infection was determined by the 2^−ΔΔ^*^CT^* method. Error bars represent the standard error of the mean (SEM) of results from 3 biologically independent experiments. *, *P < *0.05 (determined by one-way ANOVA adjusted for multiple comparisons by Dunnett’s *post hoc* test). Download FIG S5, PDF file, 0.09 MB. Copyright © 2022 Chiok et al. 2022 Chiok et al. https://creativecommons.org/licenses/by/4.0/ This content is distributed under the terms of the Creative Commons Attribution 4.0 International license.

Autophagy is characterized by degradation of the autophagy flux marker p62 within active autophagic compartments, and consequently, decreasing levels of p62 indicate functional autophagy (20). Therefore, we next evaluated p62 protein levels in RSV-infected cells. p62 protein levels decreased gradually and significantly (*P < *0.05) over the course of RSV infection (Fig. 4C and D). These results show that RSV augments Beclin1 levels, a key proautophagy mediator, and engages functional autophagy during infection.

### NS2 stabilizes Beclin1 protein.

**FIG 5:**
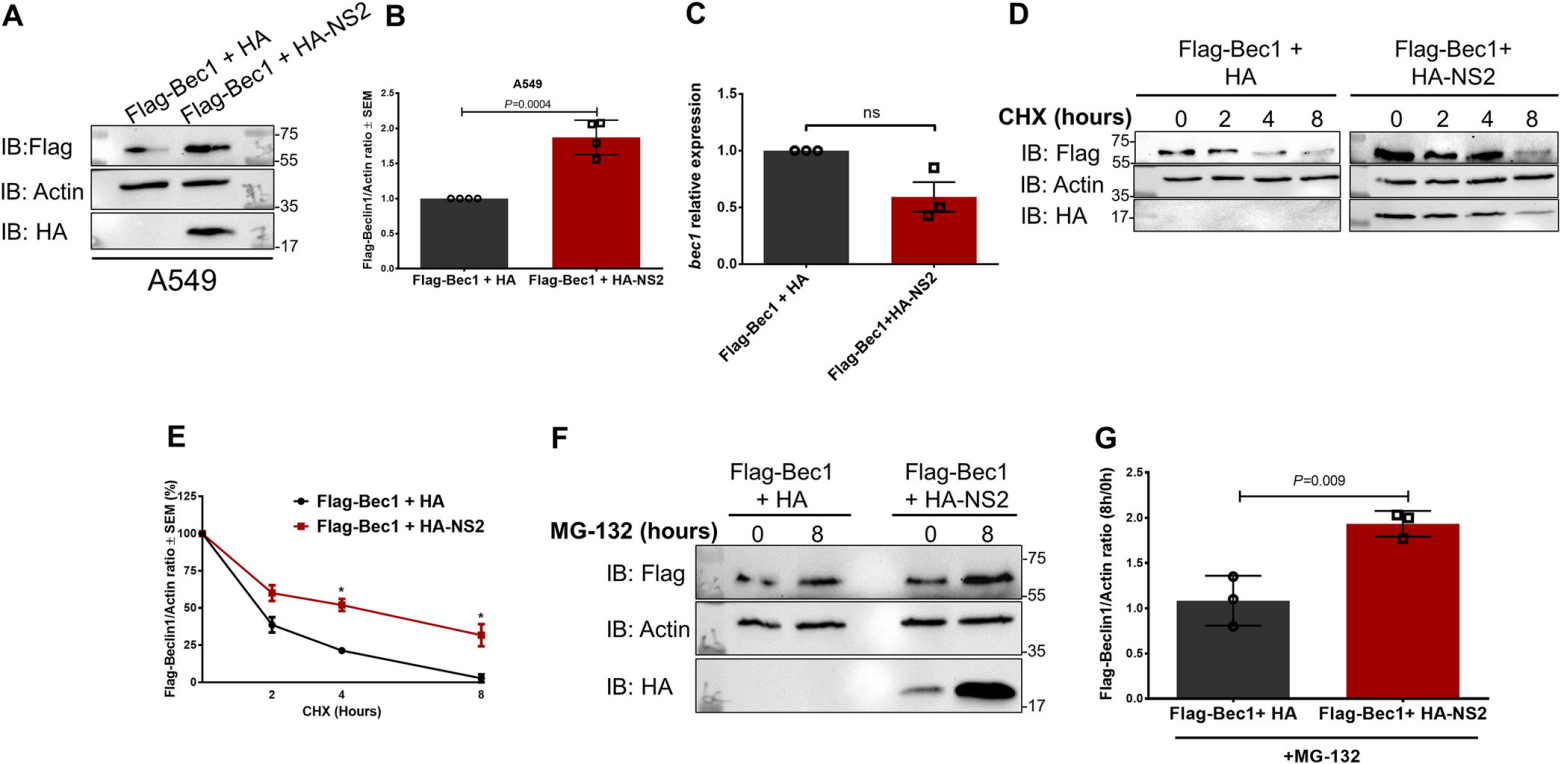
RSV NS2 protein stabilizes Beclin1 protein. (A) A549 cells were cotransfected with Flag-tagged Beclin1 (Flag-Bec1) and HA-tagged NS2 (HA-NS2) or empty HA vector control (HA). Flag-Bec1 and HA-NS2 levels were determined in cell lysates by immunoblotting (IB) using anti-Flag and anti-HA antibodies, respectively. (B) Densitometry analysis of Flag-Beclin1 levels relative to actin (Flag-Beclin1/actin) in A549 cells coexpressing Flag-Bec1 and HA-NS2. (C) RNA from A549 cells cotransfected with Flag-tagged Beclin1 (Flag-Bec1) and HA-tagged NS2 (HA-NS2) or vector control (HA) were subjected to RT-qPCR to determine *beclin1* transcript levels. Average expression relative to that of empty HA control vector (HA) was determined by the 2^−ΔΔ^*^CT^* method. (D) A549 cells coexpressing Flag-Bec1 and HA-NS2 or empty vector control (HA) were treated with the protein synthesis inhibitor cycloheximide (CHX; 75 μg/mL) for the indicated times. Flag-Bec1 and HA-NS2 levels were determined in cell lysates by IB using anti-Flag and anti-HA antibodies, respectively. (E) Densitometry analysis of Flag-Beclin1 levels relative to actin (Flag-Beclin1/actin) normalized to 0-h CHX treatment as described for panel D. (F) A549 cells coexpressing Flag-Bec1 and HA-NS2 or empty HA vector control (HA) were treated with the proteasome inhibitor MG-132 (10 μM, 8 h). Flag-Bec1 and HA-NS2 levels were determined in cell lysates by IB using anti-Flag and anti-HA antibodies, respectively. (G) Densitometry analysis of Flag-Beclin1 levels relative to actin (Beclin1/actin) in MG-132-treated A549 cells coexpressing Flag-Beclin1 and HA-NS2 as described for panel F. For each treatment (i.e., Flag-Beclin1 + HA and Flag-Beclin1 + HA-NS2), the value was calculated based on the ratio of (Flag-Beclin1/actin)_8 hpi_ to (Flag-Beclin1/actin)_0 hpi_ (where hpi is hours postinfection). Immunoblot images are representative of three independent experiments. Error bars represent the standard error of the mean (SEM) of results of 3 biologically independent experiments. *, *P < *0.05 (determined by Student’s *t* test adjusted for multiple comparisons by the Sidak-Bonferroni method). ns, not significant.

To understand the mechanisms leading to Beclin1 protein level enhancement during RSV infection (Fig. 4), we investigated whether NS2 affected Beclin1 protein stability as a result of an NS2-Beclin1 interaction (Fig. 2). First, we examined Beclin1 protein levels in A549 cells transfected with Flag-tagged Beclin1 and HA-tagged NS2 (Fig. 5A). We detected higher levels of Flag-Beclin1 protein in cells expressing NS2 following immunoblotting with anti-Flag antibody (Fig. 5A). Densitometry analysis revealed that expression of NS2 resulted in significantly increased levels of Beclin1 (*P = *0.0004) (Fig. 5B). As with RSV infection (Fig. 4A and B), Beclin1 protein levels were more than 50% higher in the presence of NS2 than in control cells (Fig. 5B). Enhanced Beclin1 protein levels were not due to upregulation of *beclin1* transcription, since expression of HA-tagged NS2 did not increase *beclin1* mRNA levels (Fig. 5C). This result indicates that NS2 coexpression leads to increased levels of Beclin1 protein at the posttranslational level.

To determine whether NS2 affects Beclin1 protein stability, we monitored Beclin1 protein decay following the addition of the protein synthesis inhibitor cycloheximide (CHX). NS2 stabilized Beclin1 protein by preventing its degradation under translation inhibition conditions (Fig. 5D). Beclin1 protein was consistently detected at higher levels in A549 cells expressing NS2, even up to 8 h of protein synthesis shutdown, than in the vector control (Fig. 5E). For instance, while Beclin1 levels decreased by 45% at 4 h of CHX treatment in the presence of NS2, a reduction of 77% occurred in the absence of NS2 (Fig. 5E). These results show that expression of the NS2 protein stabilizes Beclin1 protein by preventing its degradation.

Ubiquitin (Ub)-mediated proteasomal degradation is a major protein degradation pathway involved in Beclin1 protein turnover (35, 36). We next investigated whether NS2 affects proteasomal degradation using the proteasomal inhibitor MG-132. A549 cells coexpressing Beclin1 and NS2 or vector (control) were treated with MG-132, and the levels of Beclin1 protein were determined by immunoblotting (Fig. 5F). Beclin1 levels doubled in NS2-expressing cells treated with the proteasome inhibitor (*P < *0.05), whereas no significant change was observed in treated control cells (Fig. 5G). These results indicate that NS2 augments Beclin1 stability by diminishing proteasome-mediated Beclin1 degradation, consequently enhancing Beclin1 levels. This may constitute a mechanism utilized by NS2 to induce autophagy, since higher levels of intracellular Beclin1 protein mediate autophagy (33, 34).

### NS2 contributes to autophagy during RSV infection.

**FIG 6:**
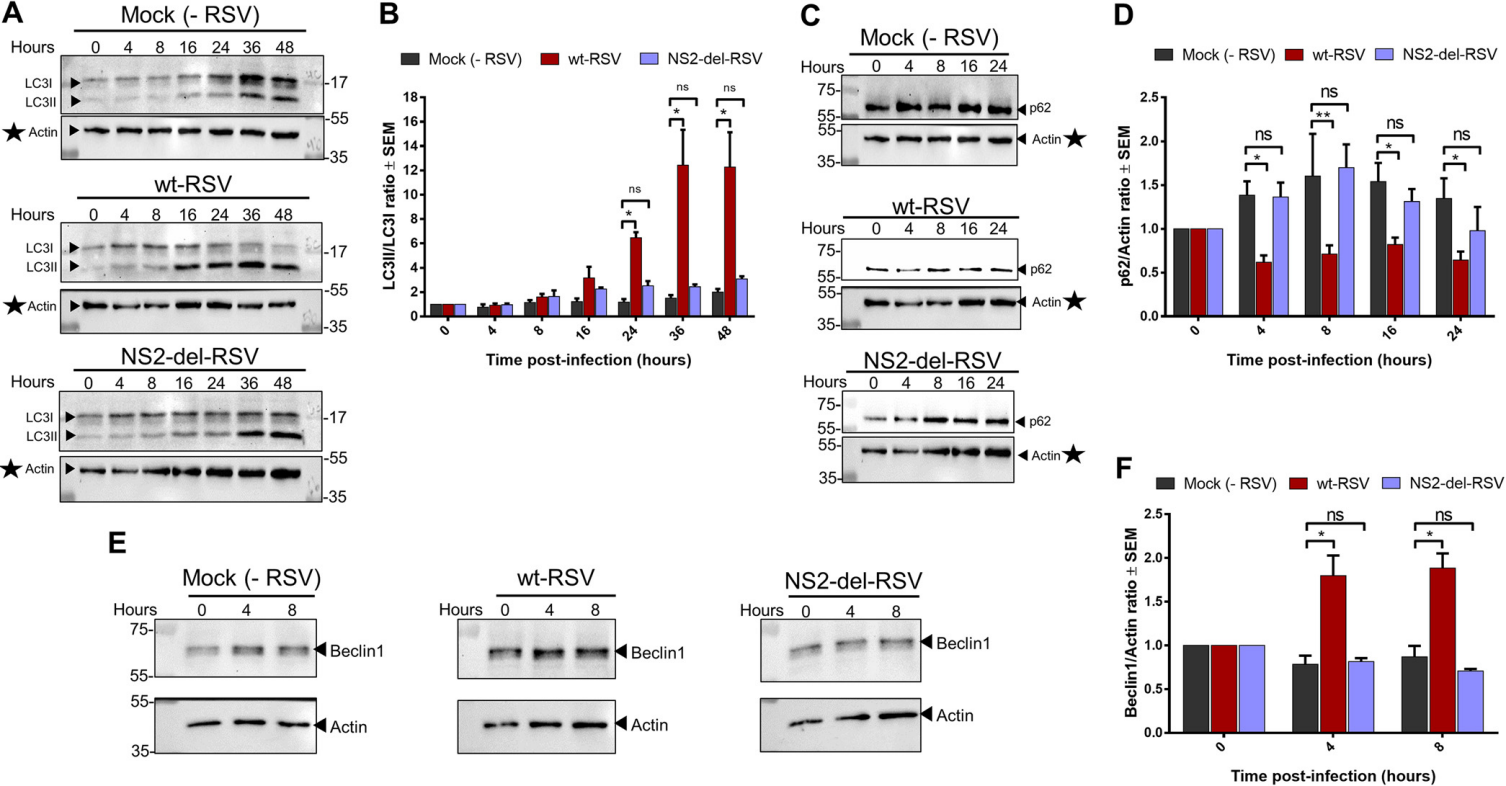
RSV NS2 protein contributes to autophagy during RSV infection. (A) Mock-infected (−RSV), wild-type RSV-infected (wt-RSV), and NS2-lacking RSV (NS2-del-RSV)-infected HEp-2 cells (MOI = 5) were subjected to immunoblotting. The autophagy status was determined by analyzing the LC3I-to-LC3II conversion via the LC3II/LC3I ratio. (B) Densitometry analysis of LC3II levels relative to LC3I (LC3II/LC3I) normalized to time 0 h following mock infection and infection with wt-RSV and NS2-del-RSV as described for panel A. (C) Mock-infected (−RSV), wt-RSV-infected, and NS2-del-RSV-infected HEp-2 cells (MOI = 5) were subjected to immunoblotting with anti-p62 antibody to determine the autophagy flux status during infection. (D) Densitometry analysis of p62 levels relative to actin (p62/actin) normalized to time 0 h following mock infection and infection with wt-RSV and NS2-del-RSV as described for panel C. (E) Mock-infected (−RSV), wt-RSV-infected, and NS2-del-RSV-infected HEp-2 cells (MOI = 5) were subjected to immunoblotting with anti-Beclin1 antibody to determine the Beclin1 protein levels during infection. (F) Densitometry analysis of Beclin1 levels relative to actin (Beclin1/actin) normalized to time 0 h following mock infection and infection with wt-RSV and NS2-del-RSV as described for panel E. Immunoblot images are representative of three independent experiments. Error bars represent the standard error of the mean (SEM) of results from 3 biologically independent experiments. *, *P < *0.05 (determined by two-way ANOVA adjusted for multiple comparisons by Dunnett’s *post hoc* test). The actin immunoblot panels corresponding to LC3 and p62 immunoblots in panels A and C, respectively, are the same (indicated by stars).

To dissect a direct role of NS2 protein in autophagy induction during RSV infection, we utilized recombinant wild-type RSV (wt-RSV) and mutant RSV from which the NS2 gene had been deleted (NS2-del-RSV). HEp-2 cells infected with either wt-RSV or NS2-del-RSV were monitored for autophagy induction by detecting conversion of nonlipidated LC3I to lipidated LC3II by immunoblotting using an anti-LC3 antibody (Fig. 6A). wt-RSV induced autophagy, as evidenced by the shift of LC3I to LC3II at 16 to 48 h postinfection (Fig. 6A). In contrast, such shifting was substantially diminished or absent following NS2-del-RSV infection, suggesting a key role of NS2 in autophagy induction during RSV infection (Fig. 6A). Densitometric analysis revealed that the LC3II/LC3I ratio increased exponentially over the course of wt-RSV infection, reaching a peak of 12-fold at 36 h postinfection (*P < *0.05) (Fig. 6B). In contrast, the LC3II/LC3I ratio increased only slightly in noninfected cells or cells infected with NS2-del-RSV (Fig. 6B). Differences in the LC3II/LC3I ratio between wt-RSV and mock controls were significant beginning at 24 h postinfection (*P < *0.05). These results show that RSV NS2 plays a role in autophagy induction during RSV infection.

In addition, we examined autophagy flux by immunoblotting wt-RSV- and NS2-del-RSV-infected cell lysates with antibody against the autophagy flux marker p62 (Fig. 6C). Densitometry analysis showed that while infection with wt-RSV led to a significant decrease in p62 protein levels (20 to 40% reduction) compared to that in mock-infected cells (*P < *0.05), such reduction did not occur in NS2-del-RSV-infected cells (Fig. 6D). These results show that RSV induced functional autophagy during infection, as shown by enrichment of the autophagosome-associated LC3II and decrease of the autophagy cargo protein p62. Moreover, the RSV NS2 protein contributes to RSV-induced autophagy, since RSV lacking NS2 failed to induce a significant enhancement of LC3II or promote the loss of p62 protein.

Expression of NS2 protein stabilized the proautophagy mediator Beclin1, leading to enhanced intracellular levels of Beclin1 (Fig. 5). Therefore, we investigated whether NS2 affects Beclin1 protein levels during RSV infection using wt-RSV and NS2-del-RSV. Endogenous Beclin1 protein levels were monitored in HEp-2 cells infected with either wt-RSV or NS2-del-RSV by immunoblotting with anti-Beclin1 antibody (Fig. 6E). Densitometry analysis showed that wt-RSV infection significantly enriched Beclin1 protein levels by 2-fold at 4 h and 8 h postinfection compared to uninfected cells (*P < *0.05), although this increase is no longer apparent at 16 h postinfection (Fig. 6F). In contrast, NS2-del-RSV infection did not significantly alter Beclin1 protein levels compared to that of mock cells at any time point postinfection (Fig. 6F). Together, these results show that NS2 is a key viral factor involved in autophagy induction during RSV infection. Furthermore, NS2 enhances Beclin1 protein levels during RSV infection as a mechanism to induce autophagy.

### NS2 impairs ISGylation of Beclin1.

We next investigated the mechanism of autophagy induction due to NS2. Protein ISGylation consists of the covalent conjugation of interferon-stimulated gene 15 (ISG15) to its protein targets (7, 8). Beclin1 is regulated by conjugation to ISG15 or ubiquitin (Ub) at Lys117, Lys263, Lys265, and Lys266. Lys63-linked polyubiquitination of Beclin1 at these sites is essential for autophagy activation (6). ISG15 competes with Ub to suppress Beclin1-mediated autophagy (6), making ISGylation a negative regulator of Beclin1-mediated autophagy. While RSV infection induces ISGylation (37), RSV must be able to overcome this “antiautophagy” effect because infection induces autophagy, as shown above (Fig. 6) and by others (37). Therefore, we examined whether NS2 affects ISGylation of Beclin1 to modulate its activation and induce autophagy.

**FIG 7:**
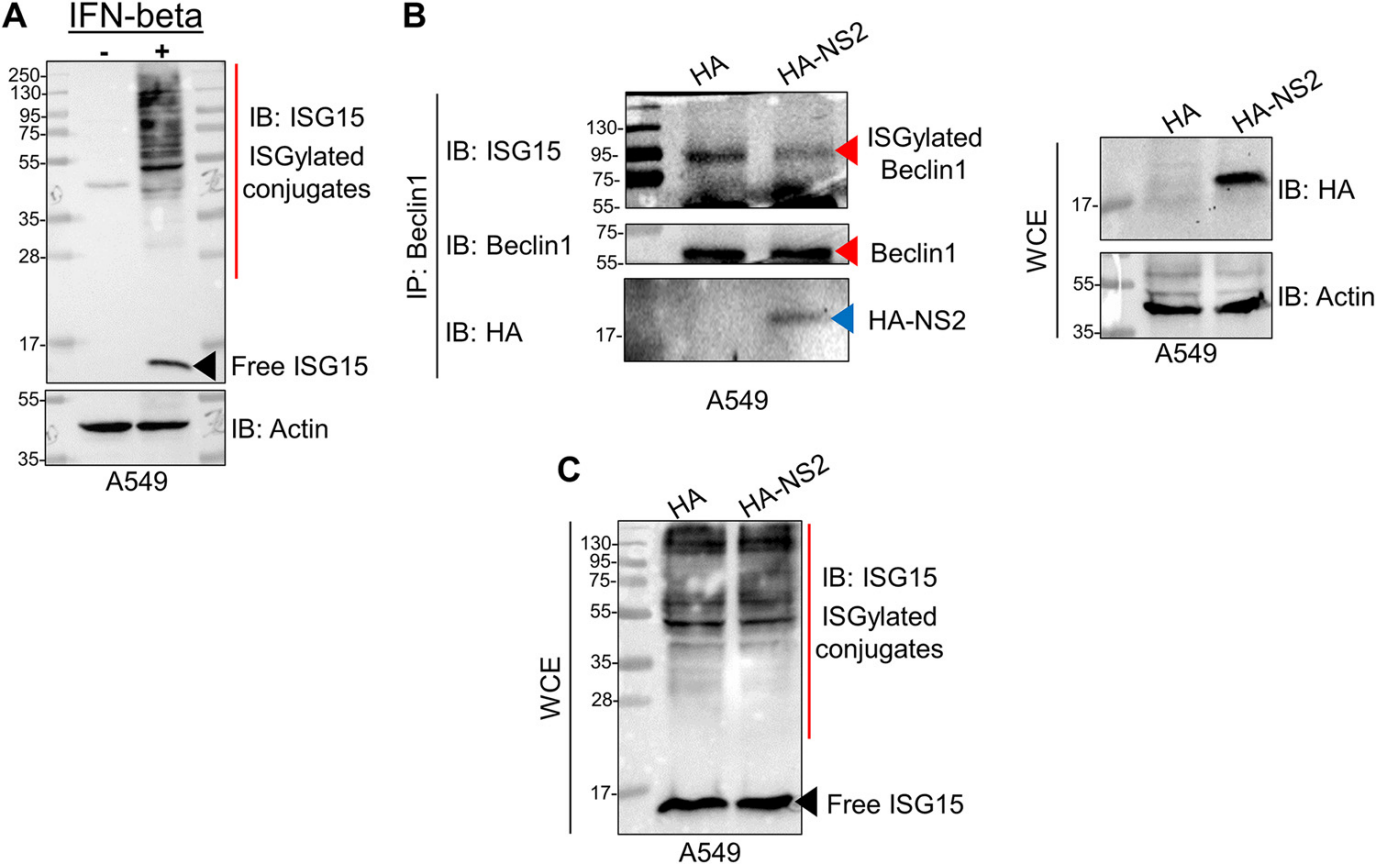
RSV NS2 protein impairs ISGylation of Beclin1 protein. (A) A549 cells were treated with interferon beta (IFN-beta, 500 U/mL) for 24 h. Whole-cell extracts (WCE) were subjected to immunoblotting (IB) with anti-ISG15 to detect ISGylated conjugates and free ISG15. A black arrowhead indicates free ISG15 protein. (B) Cell lysates collected from empty HA vector control (HA) or HA-tagged NS2 (HA-NS2) A549 cells treated with IFN-β (500 U/mL) were subjected to coimmunoprecipitation analysis to determine the ISGylation status of endogenous Beclin1. Endogenous Beclin1 was immunoprecipitated (IP) with anti-Beclin1 antibody, followed by IB with either anti-ISG15, anti-Beclin1, or anti-HA antibodies. WCE were immunoblotted with anti-HA and anti-actin antibodies. Red arrowheads indicate Beclin1 protein (ISGylated and non-ISGylated), and a blue arrowhead indicates NS2 protein. (C) WCE from panel B were subjected to IB with anti-ISG15 antibody to detect the status of ISGylated conjugates and free ISG15 following expression of HA (vector control) and HA-NS2. A black arrowhead indicates free ISG15 protein. Immunoblot images are representative of three independent experiments.

Type I interferon (e.g., IFN-β) produced during virus infection triggers ISGylation. This phenomenon is recapitulated by treating noninfected cells with IFN-β. Indeed, treatment of A549 cells with IFN-β induced ISG15 conjugate formation (Fig. 7A). To test whether NS2 affects the ISGylation of Beclin1, we treated A549 cells transfected with either HA-tagged empty vector (control) or HA-tagged NS2 with IFN-β (500 IU/mL) to induce ISGylation. These cells were subjected to coimmunoprecipitation analysis with anti-Beclin1 antibody followed by immunoblotting with anti-ISG15 antibody to determine the ISGylation status of endogenous Beclin1 (Fig. 7B). We detected a single ISGylated species of Beclin1 with a molecular weight of ∼90 kDa in both control and NS2-expressing cells treated with IFN-β (Fig. 7B). This particular ISGylated species of Beclin1 likely represents a di-ISGylated form, since unmodified Beclin1 is ∼60 kDa (Fig. 7B) and the ISG15 monomer is ∼15 kDa. Interestingly, ISGylated Beclin1 was less abundant in cells expressing NS2 than in cells expressing vector control (Fig. 7B, left panel). This NS2 effect was specific to Beclin1, as IFN-β treatment induced similar ISGylation patterns in cells expressing vector control or NS2, as shown by immunoblotting of whole-cell extracts with anti-ISG15 antibody (Fig. 7C). Moreover, diminished ISGylation of Beclin1 in the presence of NS2 is not attributable to NS2 interference with general IFN-induced ISGylation. Our results demonstrate that NS2 impairs the ISGylation of Beclin1, which may represent a mechanism to ensure the activation of available Beclin1 to induce autophagy.

## DISCUSSION

Previous studies have shown that respiratory syncytial virus (RSV) promotes autophagy in mice and in cells to modulate the innate immune and antiviral responses during infection (10, 12, 13). Autophagy also regulates RSV-associated lung pathology and pulmonary disease in infected mice (10, 12). Although autophagy plays a multifunctional role during RSV infection, the viral factor(s) that mediates such proautophagy activity remained unknown. Here, our studies identified the nonstructural protein NS2 as a viral proautophagic factor that drives RSV-induced autophagy. Our study uncovered a dual proautophagy role of RSV NS2 protein: (i) NS2 interaction with the proautophagy mediator Beclin1 results in Beclin1 stabilization, and (ii) NS2 impairs the antiautophagic effect of ISGylation on Beclin1. These two mechanisms ensure abundant availability of active Beclin1 to engage in autophagy.

Using immunoblot assays to survey the autophagy flux markers LC3II and p62, we found that RSV induces the formation and maturation of functional autophagic compartments. RSV-induced autophagy was characterized by early stabilization of the proautophagy mediator Beclin1, followed by LC3II formation and p62 reduction (Fig. 6). Additionally, the RSV protein NS2 contributed to RSV-induced autophagy, as NS2-deficient virus failed to stimulate autophagic flux or promote Beclin1 protein stabilization (Fig. 6). Coimmunoprecipitation and coimmunofluorescence assays showed that NS2 interacts and colocalizes with Beclin1 (Fig. 2), and this interaction is central to set the autophagy pathway in motion. NS2-Beclin1 binding stabilizes Beclin1, impeding its degradation by the cellular machinery (Fig. 5). Beclin1 protein levels are elevated due to NS2-mediated stabilization (Fig. 5), increasing the amount of Beclin1 available to form VPS34-VPS15-Beclin1-ATG14/UVRAG class III phosphatidylinositol 3-kinase (PI3K) complexes during autophagy initiation and maturation. Expression of NS2 protein augmented the autophagy marker LC3II and its incorporation into maturing autophagosomes as LC3II puncta, indicating a link between Beclin1 enrichment and autophagy activation (Fig. 1).

Beclin1 is subject to activating and deactivating posttranslational modifications that regulate its ability to interact with other PI3K complex proteins (reviewed in reference 38). Beclin1 ISGylation occurs due to conjugation of interferon-stimulated gene 15 (ISG15) to Beclin1 domains BH3 and CCD (6). ISGylation of Beclin1 in domains BH3 and CCD (6) inhibits autophagy by competing with Lys63 polylinked ubiquitination, which is essential for autophagy activation (6). RSV infection induces ISGylation (37) and upregulation of *ISG15* mRNA in lung epithelial cells (39), while *ISG15* mRNA is also upregulated in clinical samples from pediatric RSV patients (37). Surprisingly, our studies show that the NS2-Beclin1 interaction hampers Beclin1 ISGylation (Fig. 7), creating a pool of “hypo-ISGylated” Beclin1 to engage in active autophagy. To date, there have been no reports of viral proteins controlling ISGylation to regulate autophagy. We report a novel mechanism of virus-mediated autophagy induction in which a viral protein (i.e., RSV NS2 protein) promotes hypo-ISGylation of Beclin1 and hence its activation for the autophagic response.

Other RSV-related paramyxoviruses have evolved different strategies to manipulate the autophagy pathway by using viral proteins. The paramyxovirus measles virus (MeV) induces successive waves of sustained autophagy flux by three independent mechanisms (40, 41). One of the MeV autophagy-inducing mechanisms involves the nonstructural C protein, although the exact molecular basis remains undefined and a direct involvement of C protein in autophagy induction has yet to be established. Morbilliviruses also require interaction of the viral glycoproteins F and H with cell receptors and cell membrane fusion (i.e., syncytium formation) to induce autophagy and facilitate cell-to-cell spread (42). The NP and P proteins of Newcastle disease virus (NDV) induce autophagy through an endoplasmic reticulum (ER) stress-related unfolded protein response (PERK and ATF6 pathways) (43). In contrast, the paramyxovirus human parainfluenza virus type 3 (HPIV3) induces incomplete autophagy by blocking autophagosome-lysosome fusion via its P (phosphoprotein) protein (44). Our study revealed a viral protein (i.e., RSV NS2) interacting with Beclin1 protein to modulate its stability and activation for a productive autophagic response. To the best of our knowledge, our current study is the first report of a paramyxovirus nonstructural protein directly activating autophagy by modulating Beclin1. Furthermore, NS2 manipulation of both the autophagy and ISGylation pathways is a novel function that expands its ability to control the host response against RSV.

**FIG 8:**
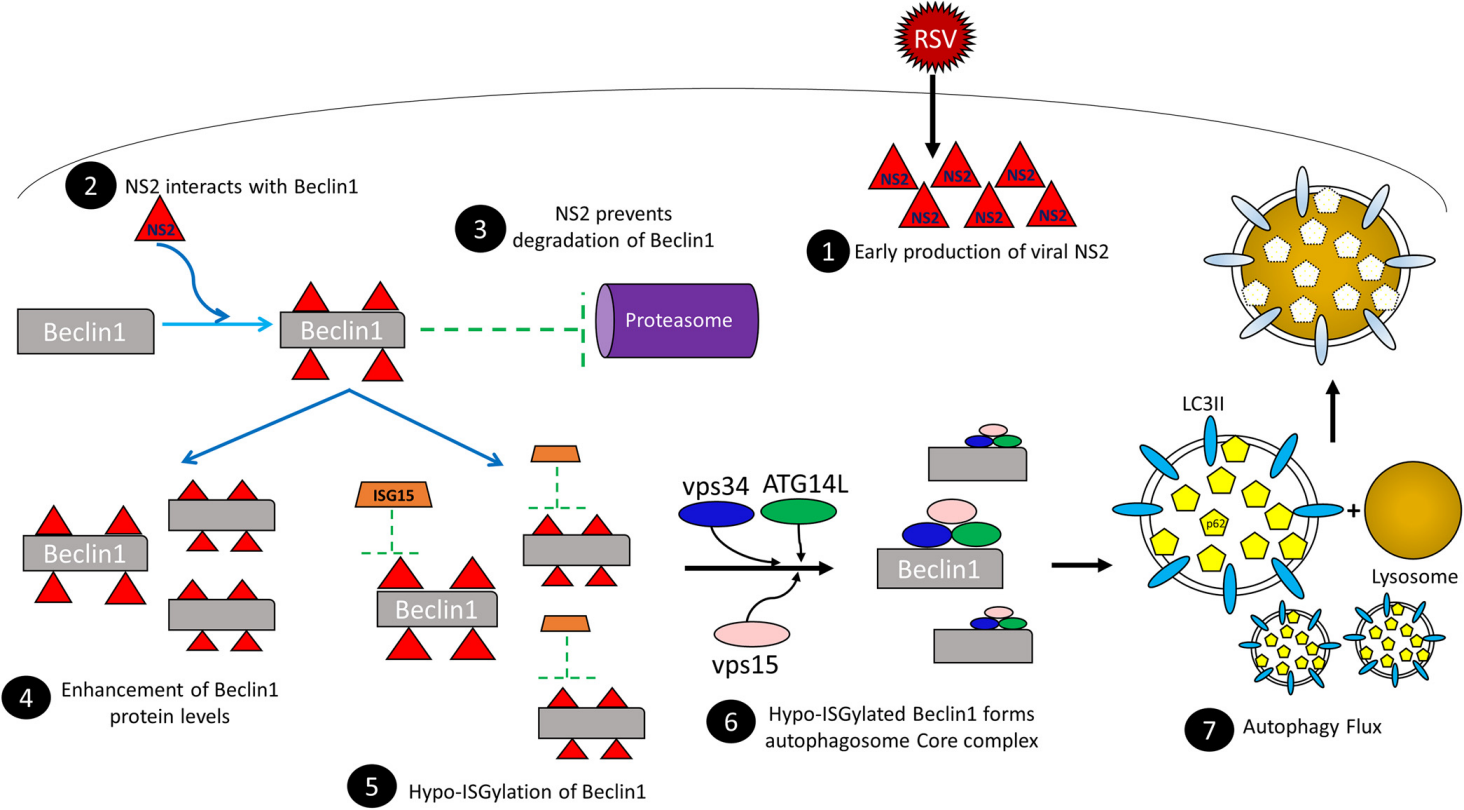
Schematic model showing RSV-induced autophagy mediated by the RSV NS2 protein. RSV NS2 protein produced shortly after virus infection (1) interacts with the proautophagy mediator Beclin1 protein (2), preventing its degradation by the proteasome (3) and resulting in enhanced Beclin1 protein levels (4). NS2 also promotes hypo-ISGylation of Beclin1 protein (5), enabling it to form a functional complex with the autophagosome core proteins comprising ATG14L, vps34, and vps15 (6). This results in autophagy flux induction (7) and autophagy.

We propose a model of the molecular mechanism underlying RSV-induced autophagy (Fig. 8). We demonstrate that the RSV nonstructural protein NS2 interacts with and stabilizes the proautophagy regulator Beclin1. The NS2-Beclin1 interaction increases Beclin1 protein levels and makes it available for autophagy by disrupting Beclin1 protein ISGylation. Autophagy likely has multifold effects in the pathobiology of RSV by regulating lung inflammation and airway disease severity (13, 45). It may also have an immune-modulatory effect in the airway by controlling the adaptive immune response in the RSV-infected respiratory tract (13, 45). Furthermore, it remains unknown whether other redundant proautophagy mechanisms take place during RSV infection or how RSV proteins escape autophagy-dependent degradation. Understanding the mechanisms governing the ability of RSV to manipulate the immune response is valuable for the development of therapeutic and prophylactic interventions targeting the host. This is of paramount importance to control viruses that insidiously hijack and deregulate innate immune antiviral responses to facilitate virus propagation.

## MATERIALS AND METHODS

### Reagents.

Cycloheximide (catalog no. 14126) was purchased from Cayman Chemical (MI, USA), and MG-132 (catalog no. tlrl-mg132) was obtained from InvivoGen (CA, USA). Rabbit anti-LC3 (catalog no. 4108S), rabbit anti-Beclin1 (catalog no. 3738S), and rabbit anti-p62 (catalog no. 8025S) were purchased from Cell Signaling Technologies (MA, USA). Rabbit anti-actin (catalog no. A300-485A) was purchased from Bethyl Laboratories (TX, USA). Mouse anti-Flag (catalog no. F1804) and mouse anti-HA (catalog no. 26183) antibodies were obtained from Sigma-Aldrich (MO, USA) and ThermoFisher Scientific (MA, USA), respectively. Fluorescein isothiocyanate (FITC) goat anti-rabbit IgG (catalog no. 111-095-003) and Alexa Fluor 594 donkey anti-mouse IgG were purchased from Jackson ImmunoResearch Laboratories (PA, USA). Anti-ISG15 antibody was generated as described previously (46). Interferon beta (catalog no. 10704-HNAS) was purchased from Sino Biological US, Inc. (PA, USA).

### Cell culture and viruses.

Cell cultures were maintained at 37°C in a 5% (vol/vol) CO_2_ atmosphere. Human lung epithelial A549 cells (ATCC; catalog no. CCL-185) and HEK293 cells (human embryonic kidney cells) (ATCC; catalog no. CRL-1573) were cultured in complete Dulbecco’s modified Eagle medium (DMEM) containing 10% (vol/vol) fetal bovine serum (FBS), 100 IU/mL penicillin, and 100 μg/mL streptomycin (Gibco). Human laryngeal epithelial HEp-2 cells (ATCC; catalog no. CCL-23) were cultured in Eagle’s minimum essential medium (EMEM) supplemented with 10% (vol/vol) FBS, 1% (vol/vol) nonessential amino acids, 100 IU/mL penicillin, and 100 μg/mL streptomycin. A549 cells stably expressing mCherry-LC3II were cultured in Roswell Park Memorial Institute (RPMI) medium supplemented with 10% (vol/vol) FBS, 100 IU/mL penicillin, and 100 μg/mL streptomycin (22). RSV strain A2 was purified as described previously (47). Recombinant RSV strain A2 lacking the nonstructural protein NS2 (NS2-del-RSV) and its wild-type counterpart (wt-RSV) were generated and purified as described previously (48, 49).

### Virus infection.

Wild-type recombinant RSV (wt-RSV) and RSV with NS2 deleted (NS2-del-RSV) have been previously described (48, 49). Virus stocks were produced and titered by plaque assay in Vero cells. For infection, subconfluent A549 cells were infected with RSV (multiplicity of infection [MOI], 1). HEp-2 cells were infected at an MOI of 5 with wt-RSV and NS2-del-RSV. RSV adsorption was performed for 1.5 h in serum-free, antibiotic-free Opti-MEM medium (Gibco). Cells were then washed with phosphate-buffered saline (PBS), and infection was continued in the presence of complete medium containing serum. Following infection, cells were washed in PBS and lysed in PBS/Triton X-100 in the presence of protease inhibitors for immunoblotting assays with antibodies.

### Cell transfection.

Cells were transfected with Lipofectamine 2000 (Invitrogen) at 80% confluence in 12-well plates with various mammalian expression plasmids: control HA vector (1 μg/well), HA-NS2 (1 μg/well), HA-NS1 (1 μg/well), Flag-Beclin1 (1 μg/well), and Flag-tagged Beclin1 deletion mutants (BH3, CCD, ECD) (1 μg/well). Transfected cells were washed with PBS before lysis in PBS/Triton X-100 in the presence of protease inhibitors for immunoblotting assays.

### CHX and MG-132 assays.

Subconfluent A549 cells were transfected with 1 μg of the indicated plasmids (control HA vector or HA-tagged NS2) for 16 h. Transfected cells were washed with PBS before treatment with cycloheximide (CHX) (75 μg/mL) or MG-132 (10 μM) prepared in complete cell medium for the indicated times. Cells were then lysed in PBS/Triton X-100 in the presence of protease inhibitors for immunoblotting assays with the indicated antibodies.

### Cell treatment with IFN-β.

Subconfluent A549 cells were treated with IFN-β (500 IU/mL) for 24 h to detect ISGylated conjugates following immunoblotting with anti-ISG15 antibody.

### Immunoblotting.

Cells lysates were subjected to SDS-PAGE and transferred to nitrocellulose membranes. Immunoblots were blocked in 5% milk in PBS-T (PBS plus Tween20) and probed with the indicated primary antibodies overnight at 4°C. Immunoblots were washed in PBS-T before incubation with the corresponding secondary antibodies. Rinsed immunoblots were developed using a Western Lightning Plus-ECL chemiluminescence kit (Perkin-Elmer, catalog no. NEL103001EA) and imaged in a Chemidoc XRS+ instrument (Bio-Rad, CA, USA). Densitometric analysis was performed using ImageLab software v5.1 (Bio-Rad, CA, USA).

### LC3II puncta detection by fluorescence microscopy.

A549 mCherry-LC3II cells were seeded onto glass coverslips and cultured for 24 h until subconfluent. Cells were infected with RSV for 16 h before the coverslips were fixed in 4% (vol/vol) paraformaldehyde. Fixed coverslips were washed in PBS and rinsed in double-distilled water before mounting onto glass slides using gold antifade mountant with DAPI (4′,6-diamidino-2-phenylindole) (ThermoFisher Scientific catalog no. P26935). Slides were imaged with a Leica microscope coupled to Leica LAS X software v3.0.11.20652 (Leica Microsystems, Inc., Germany). The number of LC3II puncta per cell was counted using the ImageJ particle analysis function (50). LC3II puncta were counted in at least 25 individual cells per experiment in two independent experiments.

### Coimmunofluorescence analysis.

A549 cells were seeded onto glass coverslips and cultured for 24 h before transfection with 1 μg of the indicated plasmids. Transfected A549 cells were cultured for 16 h, washed with PBS, and fixed with 4% paraformaldehyde. Cells were permeabilized with 0.25% (vol/vol) Triton-X in PBS, rinsed, and then blocked for 1 h in 1% (vol/vol) bovine serum albumin (BSA) in PBS. Coverslips were incubated with the indicated primary antibodies overnight at 4°C. Fluorescein (FITC)- and Alexa Fluor 594-labeled secondary antibodies were added for 1 h at room temperature before mounting onto glass slides using gold antifade mountant with DAPI (ThermoFisher Scientific catalog no. P26935). Slides were imaged with a Leica microscope coupled to Leica LAS X software v3.0.11.20652 (Leica Microsystems, Inc., Germany).

### Coimmunoprecipitation assay.

Subconfluent HEK293 or A549 cells were transfected with 4 μg of the indicated plasmids. Transfected cells were cultured for 16 h before lysis in PBS plus Triton X-100 in the presence of protease inhibitors. Cell lysates were clarified by centrifugation at 16,300 × *g* for 10 min at 4°C. Protein G agarose beads (ThermoFisher Scientific catalog no. 20398) were conjugated overnight at 4°C with 4 μg of the indicated antibodies. Clarified cell lysates were incubated overnight at 4°C with protein G agarose beads conjugated to the indicated antibodies. Agarose beads were washed in PBS/Tris-HCl (50 mM) buffer in the presence of protease inhibitors. Protein complexes bound to the agarose resin were eluted by boiling the agarose beads in SDS-PAGE sample buffer. Eluted samples were used in immunoblotting and probed with the indicated antibodies.

### Reverse transcription quantitative real-time PCR (RT-qPCR).

A549 cells were transfected with Flag-Beclin1 and with either vector HA-control or HA-NS2. RNA was extracted using TRIzol reagent (ThermoFisher Scientific catalog no. 15596026) and treated with RNase-free DNase1 (ThermoFisher Scientific catalog no. EN0525) according to the manufacturer’s instructions. Synthesis of cDNA was carried out using an Applied Biosystems high-capacity cDNA reverse transcription kit (Fisher Scientific catalog no. 43-688-14) using 500-ng DNase-treated RNA templates. qPCR was performed using SsoAdvanced Universal SYBR green supermix (Bio-Rad catalog no. 1725271), 0.2 μM forward (5′- GGTGTCTCTCGCAGATTCATC-3′) and reverse (5′- TCAGTCTTCGGCTGAGGTTCT-3′) primers targeting *beclin1*, and 0.2 μM forward (5′- GATCATCAGCAATGCCTCCT-3′) and reverse (5′- TGTGGTCATGAGTCCTTCCA-3′) primers targeting the housekeeping gene *GAPDH*. qPCRs were performed in a CFX96 Touch real-time PCR detection system instrument coupled to Bio-Rad CFX manager v3.6 (CA, USA). Samples were run in triplicate. Quantitative data were analyzed using the 2^−ΔΔ^*^CT^* method by first subtracting the threshold cycle (*C_T_*) values of *GAPDH* from the *C_T_* values of *beclin1* (Δ*C_T_*) for each sample. Next, Δ*C_T_* values of control HA-transfected samples were subtracted from the Δ*C_T_* values of HA-NS2 samples for normalization.

### Statistical analysis.

Student’s *t* test was performed to evaluate differences in fluorescence microscopy analysis for LC3II puncta quantitation. Student’s *t* test adjusted for multiple comparisons by the Sidak-Bonferroni method was performed to evaluate immunoblot densitometry analysis from two experimental groups at multiple time points. One-way analyses of variance (ANOVA) adjusted by Dunnett’s *post hoc* test were performed to evaluate immunoblot densitometry analysis from three experimental groups. Two-way ANOVA adjusted by Dunnett’s *post hoc* test were performed to evaluate immunoblot densitometry analysis from three experimental groups compared at multiple time points. qPCR data were analyzed using one-way ANOVA adjusted by Dunnett’s *post hoc* test. A *P* value of <0.05 was considered significant for all statistical tests. All statistical tests were performed using GraphPad Prism v6.01 (CA, USA).
