# Supplementary figures and images for: Human Respiratory Syncytial Virus NS2 Protein Induces Autophagy by Modulating Beclin1 Protein Stabilization and ISGylation
Source: mBio. 2022 Jan 18;13(1):e03528-21. doi: 10.1128/mbio.03528-21 (PMC8764521; doi:10.1128/mbio.03528-21)

**Fig. S1**

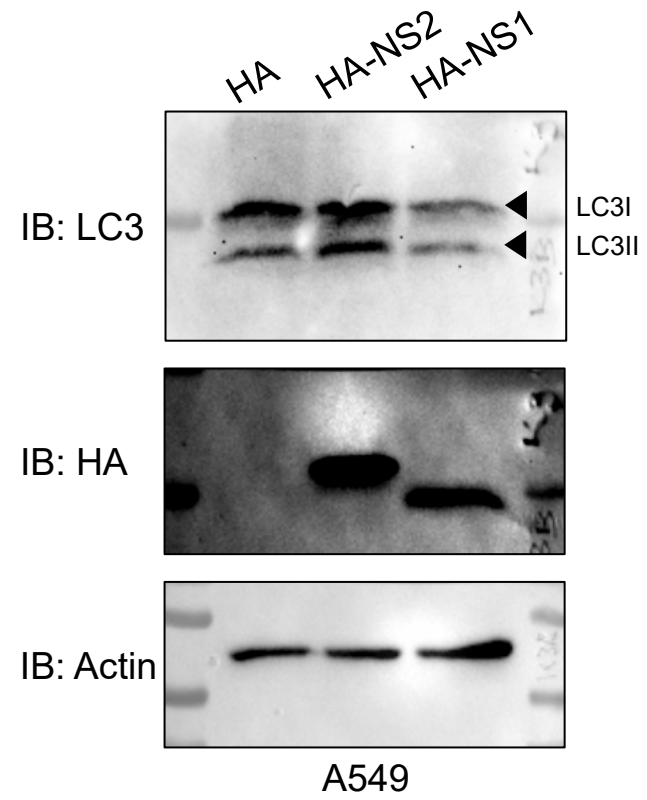

Supplement: FIG S1 [file mbio.03528-21-sf001.pdf]

**Fig. S2**

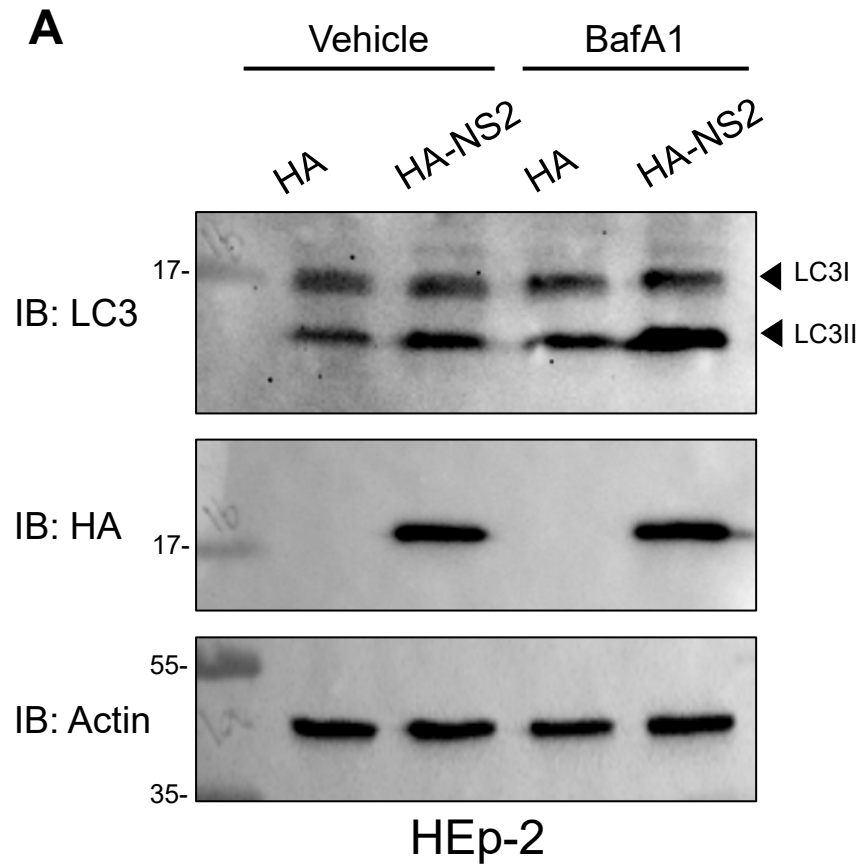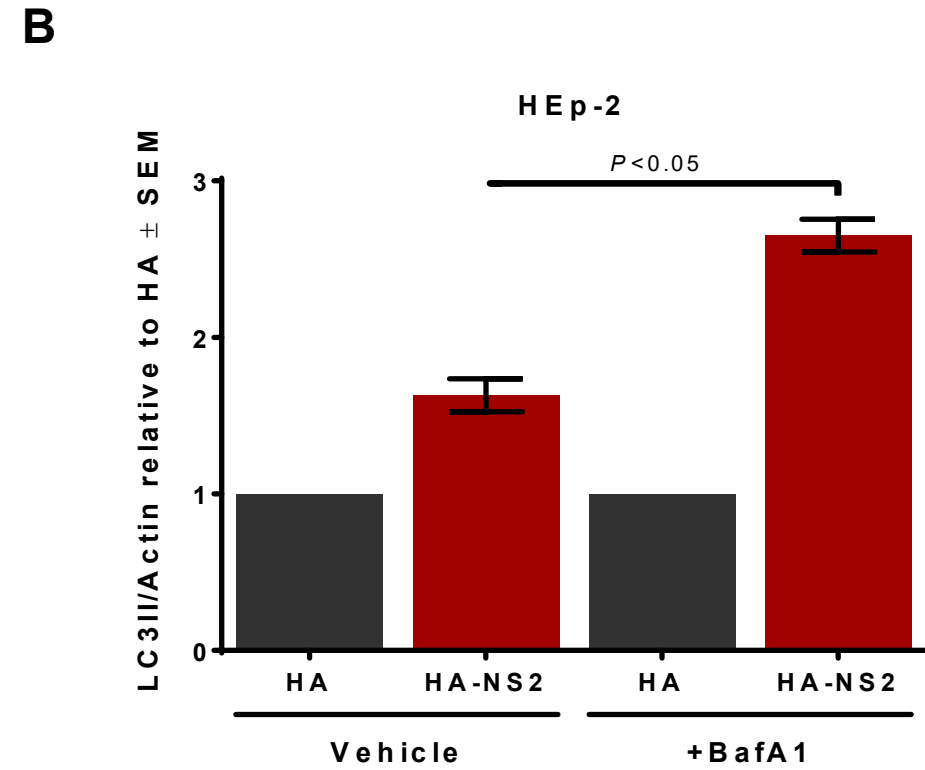

Supplement: FIG S2 [file mbio.03528-21-sf002.pdf]

**Fig. S3**

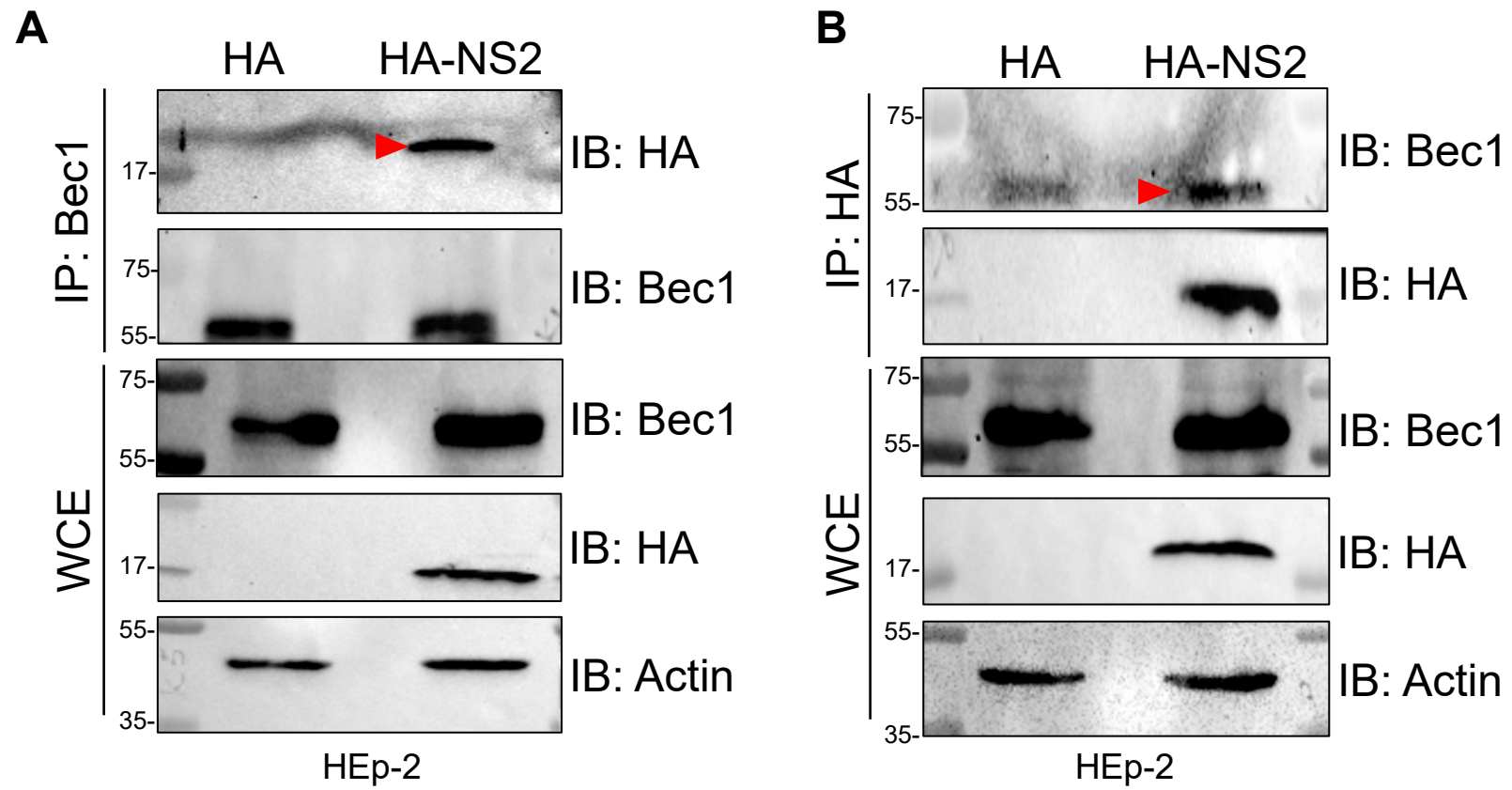

Supplement: FIG S3 [file mbio.03528-21-sf003.pdf]

**Fig. S4**

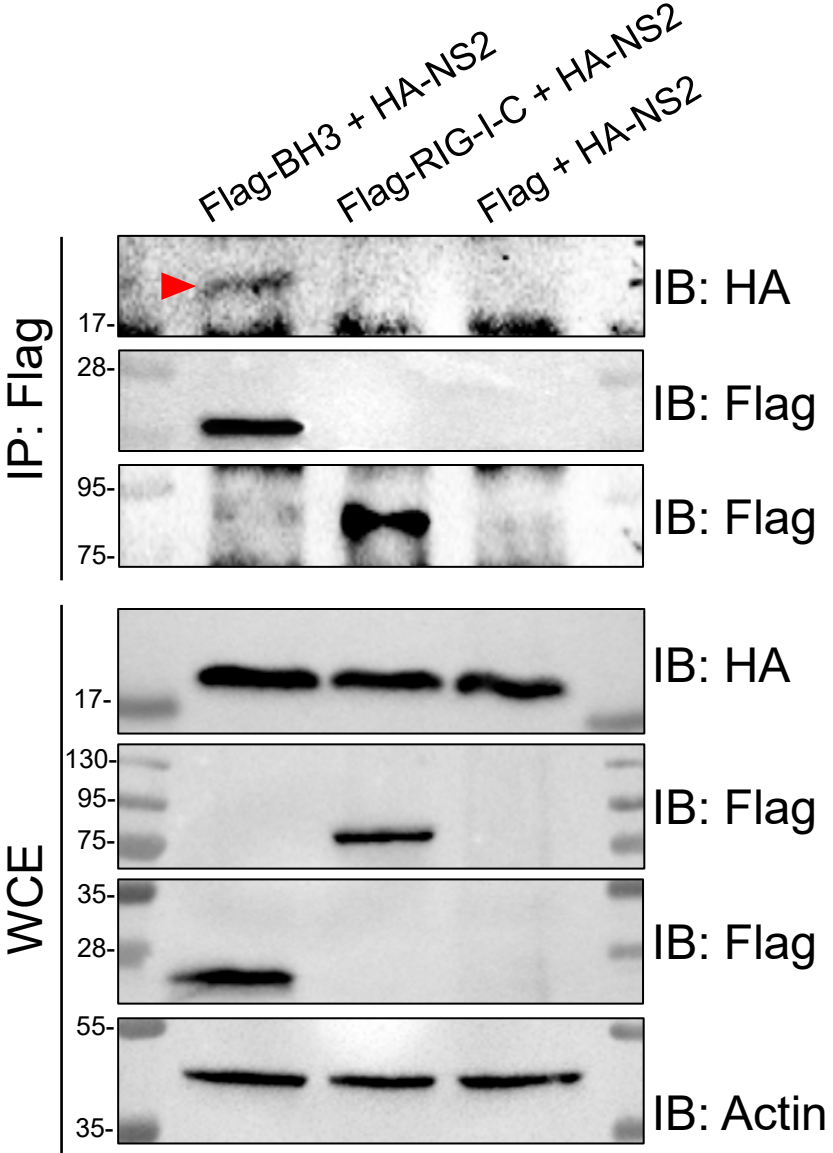

Supplement: FIG S4 [file mbio.03528-21-sf004.pdf]

Fig. S5

A

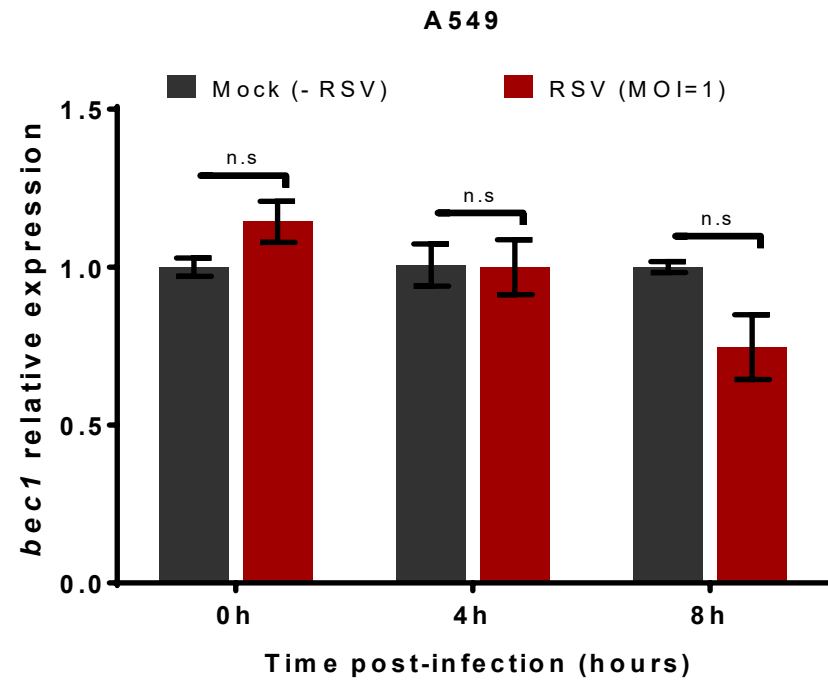

B

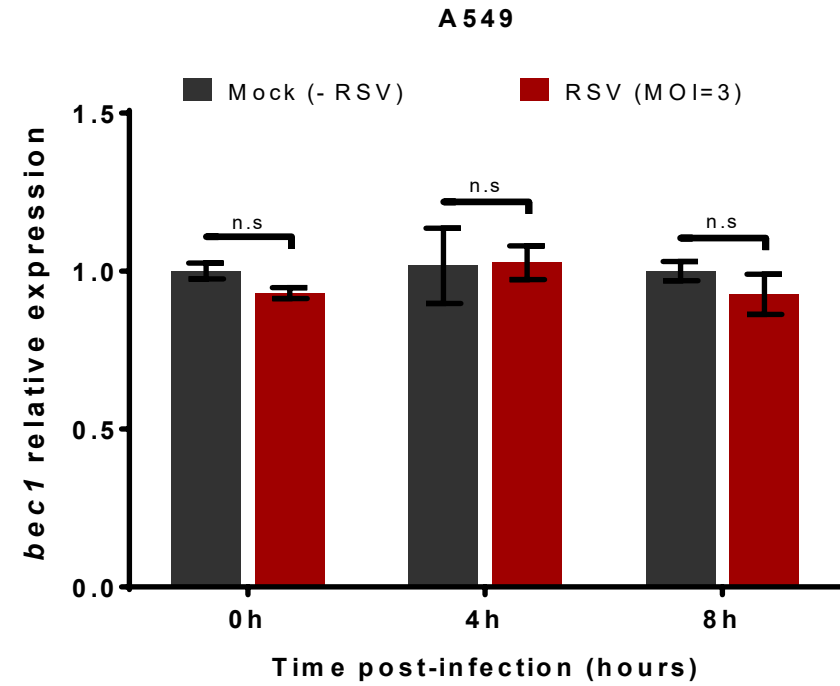

Supplement: FIG S5 [file mbio.03528-21-sf005.pdf]
